# Application of a Novel Method for Assessing Cumulative Risk Burden by County

**DOI:** 10.3390/ijerph9051820

**Published:** 2012-05-10

**Authors:** Jennifer J. Salinas, Manasi Shah, Bassent Abdelbary, Jennifer L. Gay, Ken Sexton

**Affiliations:** 1 School of Public Health, University of Texas Health Science Center at Houston, Brownsville Regional Campus, RAHC, UTB 80 Fort Brown, Brownsville, TX 78520, USA; Email: Manasi.S.Shah@uth.tmc.edu (M.S.); Bassent.E.Abdelbary@uth.tmc.edu (B.A.); Ken.Sexton@uth.tmc.edu (K.S.); 2 Health Promotion & Behavior, The University of Georgia, 330 River Road, 329 Ramsey Center, Athens, GA 30602, USA; Email: jlgay@uga.edu

**Keywords:** cumulative risk burden, socioeconomics, race/ethnicity, Texas-Mexico border, Texas

## Abstract

The purpose of this study is to apply the Human Security Index (HSI) as a tool to detect social and economic cumulative risk burden at a county-level in the state of Texas. The HSI is an index comprising a network of three sub-components or “fabrics”; the Economic, Environmental, and Social Fabrics. We hypothesized that the HSI will be a useful instrument for identifying and analyzing socioeconomic conditions that contribute to cumulative risk burden in vulnerable counties. We expected to identify statistical associations between cumulative risk burden and (a) ethnic concentration and (b) geographic proximity to the Texas-Mexico border. Findings from this study indicate that the Texas-Mexico border region did not have consistently higher total or individual fabric scores as would be suggested by the high disease burden and low income in this region. While the Economic, Environmental, Social Fabrics (including the Health subfabric) were highly associated with Hispanic ethnic concentration, the overall HSI and the Crime subfabric were not. In addition, the Education, Health and Crime subfabrics were associated with African American racial composition, while Environment, Economic and Social Fabrics were not. Application of the HSI to Texas counties provides a fuller and more nuanced understanding of socioeconomic and environmental conditions, and increases awareness of the role played by environmental, economic, and social factors in observed health disparities by race/ethnicity and geographic region.

## 1. Introduction

The cumulative impact of socioeconomic disparity has been linked to differentials in cancer, cardiovascular disease, mental health and disability [1,2,3]. The poor carry a higher burden of risk for mortality and have a shorter lifespan than those living in affluence [4]. Lifestyle and the places we live create these health and mortality inequities. The most often studied factors are health behaviors associated with greater risk of disease (*i.e*., smoking, drinking, high risk sexual behaviors, *etc*.) [5,6]. Understanding what aspects of living in poverty contribute to greater cumulative risk of disease and mortality is essential to design effective policies that narrow disparities by socioeconomic status and by race and ethnicity. While community attributes are known to be associated with health and mortality [7], little progress has been made in systematically examining environments and the accumulation of factors that affect individual and cumulative health risk at an aggregated level, such as a census tract, zip code, or county. Of course, it must be kept in mind that the true, underlying distribution of environmental stressors, vulnerability factors, and the link between them is likely to vary depending on the spatial scale of analysis and the resolution of the available data [8]. The purpose of this study is to apply the Human Security Index (HSI) as a tool to detect social, economic, and environmental cumulative risk burden at a county-level in the state of Texas. Because Texas is a large, ethnically-diverse state with a large population of socioeconomically disadvantaged people living along the border with Mexico, the setting provides a unique opportunity to apply the HSI and examine its capacity for distinguishing the relative effects of race/ethnicity and poverty.

## 2. Materials and Methods

### 2.1. The Human Security Index

The HSI is an instrument that has been adapted from the Human Development Index (HDI) [9]. The HSI was developed initially to characterize socioeconomic inequality in developed countries where poverty takes on different dimensions than in developing countries. The HSI is an index comprising a network of three sub-components or “fabrics”; the Economic, Environmental, and Social Fabrics. The Social Fabric (component) is further subdivided into the (a) Education; (b) Health; (c) Crime & Punishment and (d) Social Stress subcomponents (or subfabrics). Each of the three Fabrics (Economic, Environmental, and Social) characterizes a different aspect of quality of life, and provides an overall assessment of socioeconomic conditions. We assess each fabric individually and the overall instrument using factor analysis. In addition, we assess association of burden with ethnic concentration. We hypothesize that the HSI will be a useful instrument for identifying and analyzing socioeconomic conditions that contribute to cumulative risk burden in vulnerable communities. Moreover, we expect to identify statistical associations between cumulative risk burden and (a) ethnic concentration and (b) geographic proximity to the Texas-Mexico border.

### 2.2. Data

Data for the HSI were obtained for 254 counties in Texas. Sources of information included databases from the US Census Bureau, the Environmental Protection Agency (EPA), National Center for Health Statistics (NCHS), Health and Human Services (HHS), Behavioral Risk Factor Surveillance Survey (BRFSS) Texas Department of State Health Services (DSHS), Small Area Health Insurance Estimates (SAHIE), United States Department of Agriculture (USDA), Bureau of Labor Statistics (BLS), Local Area Unemployment (LAU) and Federal Bureau of Investigation (FBI). The variables, the year and the source institute or organization from which the data were obtained are listed in Table 1. The most recently available data, which in most cases was collected within the past 5 years, were used. In a few instances (*i.e*., % Adult Literacy (below BPLS), CO_2_ Emissions, *etc*.) the most current data were within the previous decade. Due to the fact that the components were not consistently available in the same metric (*i.e*., rates, percentages, *etc*.), logarithmic transformations were performed so that all variables were based on +/− standard deviations from their mean, which enabled us to add individual measures together to create the overall HSI score and the three individual fabric scores. A higher score for the overall HSI, the Fabrics and the Subcomponents of the Social Fabric is indicative of higher insecurity. Because in some cases higher variable scores were indicative of insecurity (*i.e.*, % poverty) and in other cases indicative of security (contrary to insecurity such as median income), all variables were recoded so that the higher scores would reflect a higher insecurity. For example, higher median income would be contrary to insecurity, so the reverse code was used to construct the Economic Fabric. Additionally, % population > 25 years with Bachelors degree would also be contrary to insecurity, so the reverse code was used to construct the Social Fabric. The 20 counties with total population of less 2,000 were omitted from the analysis, so the total number of counties analyzed was 234. Statistical analysis was performed using STATA 12 [10]. A complete list of HSI variables and sources is provided in the Appendix (see Table 1).

**Table 1:** Factor analysis results for fabric indicator.

		Eigen Value	Factor Loadings
**Economic Fabric Factor**		3.11	
	Poverty		0.94
	Unemployment		0.50
	Median Household Income		0.92
	Inequality (Gini Co-efficient)		−0.77
	% of population on Food Stamps		0.80
**Environmental Fabric Factor**		1.46	
	Particulate Days > EPA threshold		0.41
	Ozone Days > EPA threshold		0.75
	CO_2_ emissions		−0.20
	Natural Amenity Rank		0.17
	Population change 2000–2009		0.79
**Social Fabric Factor**			
***Education Subcomponent***		3.24	
	High school graduate (incl. equivalency)		
	Average Freshman Graduate Rate		0.30
	% population 25 yrs or older High school graduate (incl. equivalency)		0.96
	% population > 25 yrs some college incl. Associate Degree		0.68
	% population > 25 yrs with Bachelors degree		0.58
	%Adult Literacy (below BPLS)		0.95
	% not proficient in English		0.73
***Health Subcomponent***		2.39	
	M&F Life Expectancy at Birth		0.84
	Lesser of F or M LE change 1985–2006		−0.40
	YPLL Premature Death Rate		0.84
	% Adult obesity		0.65
	Motor vehicle mortality rate		0.60
	Chlamydia Rate		0.14
	% Adult uninsured		−0.35
	% Zip codes with Healthy Food Access outlets		0.09
***Crime Subcomponent***		1.30	
	Violent Crimes		0.82
	Property crimes per 10,000 population		−0.10
	Incarceration		0.79
***Social Stress Subcomponent***		3.02	
	% Child Poverty		0.85
	Mentally Unhealthy Days		0.59
	Teen Birth Rate		0.80
	Mortgage foreclosure Rate		0.25
	Housing vacancy rate (excl. vacation/seasonal vacation rate)		−0.12
	Inadequate Social Support (%)		0.32
	Grandparent performing parental Role %		0.60
	Creative share		0.87
	Commute Index (% Drive Alone*Commute Time)		−0.06

*Factor Analysis of the HSI:* We assess the HSI instrument using factor analysis. Employing all variables in (see Table A1 in the Appendix); we were able to identify the best indicators for each of the three main Fabrics and subcomponents through Principal Component Factor Analysis. An Eigen Value and factor loading for each Fabric and subcomponent were generated and assessed. Using a factor loading minimum of >0.5 (a conservative and robust value for consideration of inclusion) [11], we selected variables to be included in our final indices for the Fabrics and subcomponents.

Table 1 presents the results from this analysis. There was substantial variation in factor loading between Fabrics–not all individual factors loaded above the 0.5 threshold on each Fabric. The Economic Fabric had the greatest proportion of factors, with four out of five that met the threshold; followed by the Crime and Punishment subcomponent (two of three), the Educational subcomponent (five of seven), and two subcomponents of the Social Fabric. The Environmental Fabric had three of six factors loading at or above 0.5, and the Health subcomponent of the Social Fabric Factor had four of eight. The final instruments are summarized in Table 2. Some variables (e.g., insufficient social support), which had a factor score of >0.5 but had insufficient data for the counties, were omitted from the final index.

**Table 2:** Final fabric indicators for the Human Security Index (HSI) for Texas.

Factor loading >0.5	
**Economic Fabric Factor**	
	Poverty
	Unemployment
	Median Household Income
	Inequality (Gini Co-efficient)% Population on food Stamps
**Environmental Fabric Factor**	
	Ozone Days > EPA Threshold
	Population Change 2000–2009
**Social Fabric Factor**	
*Educational Subcomponent*	
	% Population > 25 yrs High School Graduate
	% Population > 25 yrs with Bachelors Degree
	% Population Adult Literacy
	% Not proficient in English
*Health Subcomponent*	
	Male and Female Life Expectancy at Birth
	Year Potential Life Lost Rate
	Adult Obesity
	Motor Vehicle mortality
*Crime and Punishment Subcomponent*	
	Violent Crime
	Incarceration Rate
*Social Stress Subcomponent*	
	Child Poverty
	Teen Birth Rate
	Insufficient Social Support
	Grandparent in Parental Role
	Creative Share Occupations

## 3. Results

### 3.1. HSI Association with Ethnicity/Race and Texas-Mexico Border Region

*High vs. Low Scoring Counties:* In the Appendix (Table A2), the highest- and lowest-scoring counties for each Fabric and subcomponent are presented with demographic characteristics from the 2010 US Census. While highest-scoring counties varied in population size, % Hispanic, % African American and % foreign born, lowest-scoring counties tended to be primarily non-Hispanic white. In addition, the highest-scoring counties had a much higher proportion of foreign born than the lowest-scoring counties on all indices. In nearly all of the Fabrics, a county along the Texas-Mexico border is the highest-scoring. However, Maverick County, which is a border county, is the lowest-scoring for the Environmental Fabric. Starr County, located on the border and recognized as one of the poorest counties in the United States [12], is the highest for Economic Fabric, Educational subcomponent of Social Fabric and overall HSI score.

*Correlation Matrix for HSI with Ethnic Concentration and Texas-Mexico Border Region:* A correlation matrix was employed using spearman’s rho (associated p values are also reported). In Table 3, correlations are presented for the HSI by race/ethnic composition. African American ethnic concentration was significantly positively associated with the Health subcomponent 0.2096 (*p* = 0.0013), the Crime subcomponent 0.3270 (*p* = 0.0000) and the total HSI 0.1367 (*p* = 0.0367). In addition, African American ethnic concentration was negatively associated with the Educational subcomponent −0.2471 (*p* = 0.0001). Hispanic ethnic concentration was associated positively with the Economic Fabric 0.4292 (*p* = 0.0000), Social Fabric 0.5827 (*p* = 0.0000), Educational subcomponent 0.8721 (*p* = 0.0000), Social Stress 0.3928 (*p* = 0.0000), and total HSI 0.3013 (*p* =0.0000). It was negatively associated with the Environmental Fabric −0.1533 (*p* = 0.0190) and Health subcomponent −0.3116 (*p* = 0.0000). Finally correlation results for immigrant concentration revealed positive associations with Economic Fabric (0.1354 (*p* = 0.0385)), Environmental Fabric (0.1969 (*p* = 0.0025)), Social Fabric (0.3372 (*p* = 0.0000)) and Total HSI (0.4231 (*p* = 0.0000)). Immigrant concentration was negatively associated with the Health Subcomponent (−0.3756 (*p* = 0.0000)).

**Table 3:** Correlation matrix for HSI fabrics and subcomponents with county-level African American and Hispanic ethnic concentration.

	African American	Hispanic	Immigrant
**Economic Fabric**	0.0865 (0.1873)	**0.4292 (0.0000)**	**0.1354 (0.0385)**
**Environmental Fabric**	0.0851 (0.1944)	**−0.1533 (0.0190)**	**0.1969 (0.0025)**
**Social Fabric**	0.0451 (0.4919)	**0.5827 (0.0000)**	**0.3372 (0.0000)**
Education Subcomponent	**−0.2471 (0.0001)**	**0.8721 (0.0000)**	**0.6996 (0.0000)**
Health Subcomponent	**0.2096 (0.0013)**	**−0.3116 (0.0000)**	**−0.3756 (0.0000)**
Crime Subcomponent	**0.3270 (0.0000)**	0.0907 (0.1667)	0.0593 (0.3666)
Social Stress Subcomponent	−0.0096 (0.8840)	**0.3928 (0.0000)**	0.0878 (0.1805)
**Total HSI**	**0.1367 (0.0367)**	**0.3013 (0.0000)**	**0.4231 (0.0000)**

### 3.2. Identification of Regional or Geographic Trends

**Figure 1:**
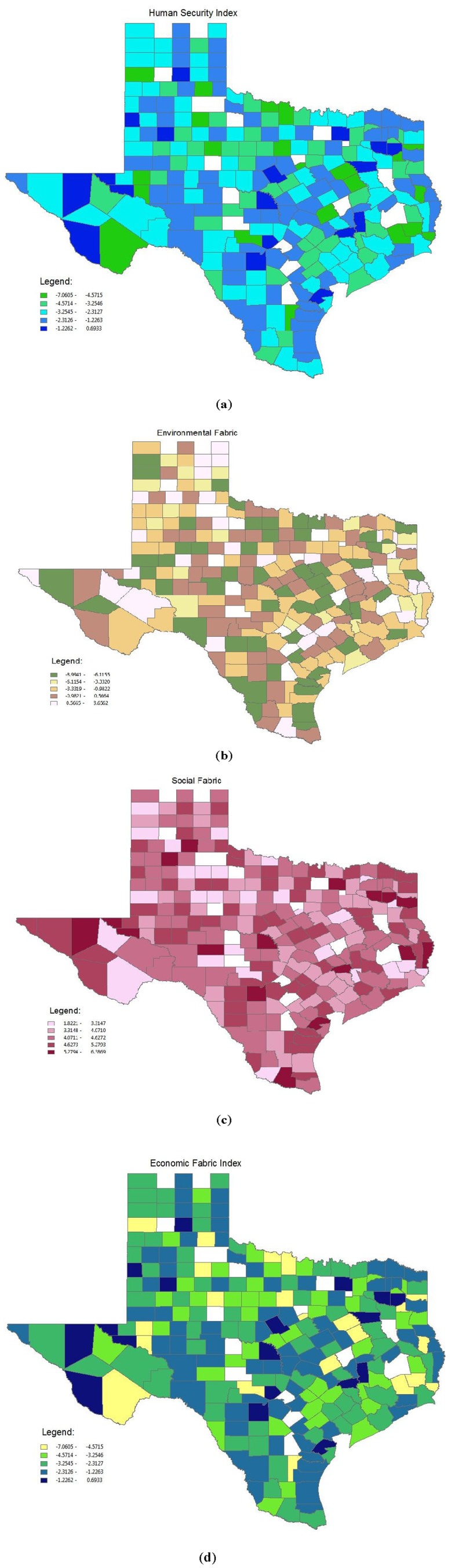
(**a**) Total Human Security Index by Texas county; (**b**) Environmental fabric component by Texas county; (**c**) Social fabric component by Texas county; **(d**) Economic fabric component by Texas county.

*Mapping with GIS:* We used GIS to provide a graphic description of the distribution of each Fabric and overall score by county in the state of Texas. Results from this analysis are shown in Figure 1. In general, scores for the overall HSI and the major Fabrics were medium to high for most of Texas. There is low overall environmental burden, but high economic and social burden for most counties in Texas. Nevertheless, as can be seen in Figure 1, contrary to what we might have expected given the extensive documentation of high poverty and relatively poor health along the Texas-Mexico border, there was no consistent pattern of higher scores along the border for the total HSI or individual Fabrics.

## 4. Discussion

The purpose of this study was to apply a novel tool that describes cumulative risk burden at a county level in Texas, and to determine its association with race/ethnic concentration and proximity to the US-Mexico border. This analysis identified those aspects of the Economic, Environmental and Social Fabrics of a county that provide the best description of cumulative risk burden. Although there is compelling evidence to suggest that poverty is associated with rates of disease and mortality [13], the conceptualization of poverty has often been linear, based on measures of income or education, and has not acknowledged variations that may exist in different contexts [14]. Socioeconomic risk is often measured in terms of percentage of poverty or income levels of a community. While this metric provides some insight into disparities, it does not provide a comprehensive assessment of the multiple mechanisms leading to disparities in community health. Using the HSI to assess socioeconomic aspects of Texas counties, we were able to identify factors most associated with economic, environmental, and social risk burden. The HSI produced more precise metrics to characterize economic, environmental and social cumulative risk, which can now be tested in other states or geographical locations and used to determine specific associations with health and mortality outcomes. It is important to keep in mind that we did not test this instrument on individual outcomes, which constrains our ability to make assertions about causal relationships. Moreover, these findings are limited to the state of Texas, and the nature and strength of observed relationships might change in other states and regions.

Attributes of economically disadvantaged environments are often assumed to be heterogeneous, which fails to recognize geographical or regional variations [14]. It is these geospatial variations in social determinants that may contribute to inequity in health outcomes [15]. Furthermore, geographical location in and of itself may limit access to resources necessary for economic growth, thereby creating a stagnant economic environment relative to others [16]. While individually a variety of factors create increased risk of disease, the combined influence of a diversity of environmental, economic, and social variables creates an elevated cumulative risk burden at both the individual and community level for those living in poverty [17].

Though many Texas border counties have median incomes among the lowest in the US [12], this analysis did not find a consistent pattern of higher cumulative risk burden along the US-Mexico border in Texas. The border region did not have consistently higher total or individual fabric scores as would be suggested by the high disease burden and low income in this region. One possible explanation for this finding is that counties with the lowest burden tended to be small and primarily non-Hispanic white. The inconsistencies in population size of counties along the border may have contributed to these findings. Therefore, explaining trends in mortality and morbidity data by socioeconomic burden or ethnic concentration may necessitate more complex analysis, such as interaction or path analysis of multiple variables, to fully appreciate the complex relationship between county-level cumulative risk and disease outcomes.

The impact of residential inequality by race and ethnicity has been extensively researched in terms of economic mobility and quality of life [18,19] with high ethnic concentration of African Americans and Hispanics tending to have lower overall levels of education, wealth and purchasing power [17]. These segregated communities lack access to quality healthcare, nutritious foods, usable parks, and in some cases, adequate infrastructure for safe drinkable water or reliable plumbing [20]. Communities that are primarily African American typically have higher rates of disease, untreated conditions and mortality than ethnically mixed or predominantly non-Hispanic white communities [21]. African Americans have lower life expectancy and carry a greater disease burden than Hispanics [22]. Hispanics have health profiles similar to or better than non-Hispanic whites.

Similarly, racial isolation or segregation is often a consequence and cause of socioeconomic inequality in neighborhoods [17]. Best documented in African Americans in northern cities such as Chicago, Detroit and Philadelphia [23,24], the highest socioeconomic disadvantage among African Americans is found in “hyper-segregated neighborhoods” or neighborhoods with the highest number of African Americans and furthest away from non-Hispanic whites [17] The consequence on health in these neighborhoods has been profound [25]. Cities that have the highest racial segregation of African Americans have higher rates of mortality and higher rates of chronic disease in adults [26,27,28].

Despite the greater likelihood of Mexican Americans living in poor neighborhoods, higher Mexican American racial isolation has not been observed to have the same negative effects on health as has been observed in African American communities. Although Mexican Americans who live in more economically disadvantaged neighborhoods are more likely to rate their health as poor, those who live in areas populated with a greater proportion of residents of Mexican origin have been observed to assess their health more favorably [29]. This apparent “protective effect” of the Mexican American neighborhood extends to cognition and mental health decline and has been observed in cancer as well [30,31]. The disjuncture of the Mexican American healthy enclave effect is in the Texas-Mexico border where diabetes and its related conditions are the prevailing public health problems. The prevalence of diabetes on the border is higher than what has been observed nationally and the incidence of new cases continues to rise. What is currently lacking in the scientific knowledge base is an adequate understanding of to what extent Mexican American ethnic enclaves may protect its residents from some diseases, while at the same time contributing to the risk of others.

In this study we examined why these two groups may differ in health outcomes by analyzing the strength of association between county-level ethnic concentration and Environmental, Economic, and Social Fabrics. While Economic, Environmental, and Social Fabrics (including the Health subcomponent) were highly associated with Hispanic ethnic concentration, the overall HSI score and Crime subcomponent were not. Moreover, while the Education Fabric and the Health and Crime subcomponents were associated with African American racial composition, Environment, Economic and Social Fabrics were not.

We found a negative association between Hispanic ethnic concentration and the Health subcomponent of the Social Fabric. This is consistent with what has been previously found with respect to Hispanic ethnic concentration in neighborhoods [31,32], and indicative of an alternative influence on health outcomes in the places that Hispanics live that is other than poverty. Like African Americans, Hispanics are more likely than non-Hispanic whites to live in poverty [18]. Nevertheless, Hispanics have health and mortality outcomes more similar to non-Hispanic whites than blacks [32]. In fact, higher Hispanic ethnic concentration is associated with lower all-cause mortality, disability, mental illness and certain forms of cancer [31]. Future research using the HSI should explore these relationships further using health outcomes to provide greater insight into the relationship between ethnic concentration and health. This is particularly important for Hispanics given their unexplained health and mortality profile [32].

An additional assessment of immigrant composition in this analysis provided some insight into the relationship between cultural environment and socioeconomics. It is often noted that immigrant communities promote better health behaviors and therefore reduces the risk of disease in its residents and offset the negative health effects of living in poverty [33,34]. Moreover, the immigrant effect is often cited as a potential explanation for the health benefits of living in Hispanic communities [33]. In a state like Texas, where the majority of immigrants do come from Latin American countries, primarily Mexico, we would expect to see patterns of association with the HSI and the individual fabrics to be similar for immigrant concentration as for Hispanic concentration. The findings from this study revealed mostly similar associations with the HSI instrument and the fabrics, except for the Environmental Fabric and the Social Stress Subcomponent of the Social Fabric. In fact the association with the Environmental Fabric is in different directions. While these associations are preliminary and more in depth analysis is required to confirm these differences, this may be indicative of independent effects of Hispanic concentration and immigrant concentration that may produce differential cumulative risk burden for disease.

The study of cumulative risk burden of places where people live is a growing field of public health, as social determinants are consistently shown to have a direct impact on disease and mortality disparities in the United States [34]. As this area of research evolves, it is increasingly apparent that choosing a particular geospatial unit of analysis can affect statistical links between (a) living environment and (b) health and mortality outcomes. How we define “community” drives our results and the subsequent conclusions that we make [8,35]. In this study, we use county as our unit of analysis, since administrative data is often aggregated to the city or county level, thereby facilitating an analysis with detailed and rich data sets. This analysis provides stable results, but at the risk of making overt assumptions about a potentially diverse group of people living in a relatively large geographic area-county.

Aggregating at a county level may disguise relationships within or between counties that may or may not be present at a smaller scale (*i.e*., zip code or census tract). For example in South Carolina, researchers found an association between the presence of toxic waste sites and population characteristics at a county level that were not present or counter to what is generally accepted in the literature [35]. Percent white was associated with a higher, not a lower, number of toxic waste sites at a county-level, and at a census tract or zip code level there was not a significant association by racial composition. The relationships observed in the current study at the county level may not be present or be different at a smaller scale, such as census tract or zip code. This emphasizes the need to conduct further analysis with aggregated data at both large-scale (county) and small-scale (census tract, zip code, *etc*.) using standardized instruments, such as the HSI, to fully evaluate the relationship between racial/ethnic composition and socioeconomic status at differing geospatial levels of resolution.

Another potential limitation of this study is the assumption that counties are mutually exclusive and that the socioeconomic conditions of adjacent counties are not interrelated [34]. While the intent of this analysis was to evaluate a standardize instrument that may be a useful way of assessing cumulative risk burden for diseases, and to assess its correlation with ethnic composition of counties, we did not take into consideration the interrelationship of counties in places like the Texas-Mexico border region. While some counties demonstrated lower-than-expected human insecurity, the influence of indirect effects of neighboring counties was not assessed. Because of the limitations of this study, it is clear that more in depth analysis of the utility of this instrument is needed.

## 5. Conclusions

Application of the HSI to Texas counties provides a fuller and more nuanced understanding of variations in socioeconomic and environmental conditions and provides insight into the role played by environmental, economic, and social factors in observed health disparities by race/ethnicity and geographic region. Our analysis identified key publicly-available descriptors of economic, environmental and social conditions and examined correlations with race/ethnicity and location. Informed risk management decisions about protecting and enhancing the health of socioeconomically disadvantaged environments necessarily depend on timely and accurate information about cumulative impacts from multiple economic, environmental, and social stressors. The HSI provides a potentially useful tool for aggregating the combined effects of multiple chemical and nonchemical stressors on community health, and apportioning the relative contribution of these factors. Future research should focus on refining and extending our analysis as well as applying the HSI in other settings and circumstances.

## Appendix A

**Table A1:** Human Security Index (HSI) variables and sources.

Variable	Source	Component
Poverty	SAIPE 2009	Economic Fabric Index
Unemployment	BLS LAU 2010	
Median Household Income	Census 2008	
Inequality (Gini Co-efficient)	Burkey, ncat.edu from 2000 Census household income	
% of Population on Food Stamps	ERS/USDA 2007	
Particulate days > EPA Threshold	CDC-Environmental Protection Agency (EPA) Collaboration 2006	Environmental Fabric Index
Ozone Days > EPA Threshold	CDC-Environmental Protection Agency (EPA) Collaboration 2006	
CO2 Emissions	Vulcan Project 2002	
Natural Amenity Scale	Modified from USDA ERS	
Natural Amenity Rank	Modified from USDA ERS	
Population Change 2000–2009	Census 2010	
		Social Fabric Index
High School Graduate (incl. GED)	US Census 2010	Education subcomponent
Average Freshman Graduate Rate	National Center for Education Statistics 2006–2007	
% Population 25 yrs or Older High School Graduate (incl. GED)	US Census 2010	
% Population > 25 yrs Some College Incl. Associate Degree	US Census 2010	
% Population > 25 yrs with Bachelors degree	US Census 2010	
%Adult Literacy (below BPLS)	National Center for Education Statistics, National Assessment of Adult Literacy 2003	
% Not Proficient in English	US Census 2008	
M&F Life Expectancy at Birth	HHS 2010	Health subcomponent
Lesser of F or M LE change 1999–1983	Ezzati *et al*. PLoS medicine	
YPLL Premature Death Rate	National Center for Health Statistics (NCHS) 2005–2007	
% Adult Obesity	National Center for Chronic Disease Prevention and Health Promotion 2008	
Motor Vehicle Mortality Rate	NCHS 2001–2007	
Chlamydia Rate	Texas DSHS 2010	
Adult uninsured	SAHIE 2007	
Adult insured (<200% of poverty level)	SAHIE 2007	Health subcomponent
% Zip codes with Healthy Food Access outlets	Census Zipcode business Patterns 2008	
Violent Crimes	Uniform Crime Reporting, Federal Bureau of Investigation 2010	Crime and punishment subcomponent
Property crimes per 10000 population		
Incarceration	Census 2010	
% Child Poverty	SAIPE 2009	
Mentally Unhealthy Days	BRFSS 2003–2009	Social Stress subcomponent
Teen Birth Rate	NCHS 2001–2007	
Mortgage foreclosure Rate		
Housing vacancy rate (excl. vacation/seasonal vacation rate)	US Census 2010	
Inadequate Social Support (%)	BRFSS 2005–2009	
Grandparent performing parental Role %	US Census 2010	
Employed in creative class occupations	USDA ERS 2003	
Creative share	USDA ERS 2003	
Commute Index (% Drive Alone*Commute Time)	ACS 5 Year Estimates 2005–009	

**Table A2:** Demographic characteristics of highest and lowest scoring by individual fabric, subcomponent and total HSI.

	Economic Fabric		Environmental Fabric		Social Fabric		Educational Subcomponent		Health Subcomponent		Crime Subcomponent		Social Stress Subcomponent		Total HSI	
	Highest	Lowest	Highest	Lowest	Highest	Lowest	Highest	Lowest	Highest	Lowest	Highest	Lowest	Highest	Lowest	Highest	Lowest
	Starr *	Williamson	Titus	Maverick *	Brooks *	Collin	Starr *	Marion	Wheeler	Presidio	Potter	Shackelford	Zapata *	Collin	Starr *	Carson
Population Size	60,968	422,679	32,334	54,258	7,223	782,341	60,968	10,546	5,410	7,818	121,073	3,378	14,018	782,341	60,968	6,182
% Hispanic	95.5%	23.2%	39.6%	95.7%	91.2%	14.7%	95.5%	3.1%	24.8%	83.4%	35.3%	10.1%	93.3%	14.7%	95.5%	8.5%
%African American	0.1%	6.2%	9.6%	0.2%	0.5%	8.5%	0.1%	22.0%	2.1%	0.6%	10.2%	0.9%	0.1%	8.5%	0.1%	0.6%
Foreign Born	30.0%	10.3%	19.3%	34.2%	4.4%	17.2%	30.0%	2.3%	7.4%	28.1%	13.2%	2.9%	26.1%	17.2%	30.0%	2.0%

* Border County.
